# The anticancer activity of bile acids in drug discovery and development

**DOI:** 10.3389/fphar.2024.1362382

**Published:** 2024-02-20

**Authors:** Weijian Li, Lu Zou, Shuai Huang, Huijie Miao, Ke Liu, Yajun Geng, Yingbin Liu, Wenguang Wu

**Affiliations:** ^1^ Department of Biliary-Pancreatic Surgery, Renji Hospital Affiliated to Shanghai Jiao Tong University School of Medicine, Shanghai, China; ^2^ Shanghai Key Laboratory of Biliary Tract Disease Research, Shanghai, China; ^3^ State Key Laboratory of Oncogenes and Related Genes, Shanghai, China; ^4^ Shanghai Research Center of Biliary Tract Disease, Shanghai, China

**Keywords:** bile acid biosynthesis, primary bile acid, secondary bile acid, anticancer activity, bile acid derivatives

## Abstract

Bile acids (BAs) constitute essential components of cholesterol metabolites that are synthesized in the liver, stored in the gallbladder, and excreted into the intestine through the biliary system. They play a crucial role in nutrient absorption, lipid and glucose regulation, and the maintenance of metabolic homeostasis. In additional, BAs have demonstrated the ability to attenuate disease progression such as diabetes, metabolic disorders, heart disease, and respiratory ailments. Intriguingly, recent research has offered exciting evidence to unveil their potential antitumor properties against various cancer cell types including tamoxifen-resistant breast cancer, oral squamous cell carcinoma, cholangiocarcinoma, gastric cancer, colon cancer, hepatocellular carcinoma, prostate cancer, gallbladder cancer, neuroblastoma, and others. Up to date, multiple laboratories have synthesized novel BA derivatives to develop potential drug candidates. These derivatives have exhibited the capacity to induce cell death in individual cancer cell types and display promising anti-tumor activities. This review extensively elucidates the anticancer activity of natural BAs and synthetic derivatives in cancer cells, their associated signaling pathways, and therapeutic strategies. Understanding of BAs and their derivatives activities and action mechanisms will evidently assist anticancer drug discovery and devise novel treatment.

## 1 Introduction

Bile acids (BAs) are physiological metabolites that are synthesized in the liver, stored in the gallbladder, and excreted into the intestine through the biliary system (Chiang and Ferrell, 2019). BAs participate in the nutrient absorption and secretion, and regulate lipids and glucose metabolism, thus maintaining metabolic homeostasis (Collins et al., 2023). Although BAs regulate intestinal flora growth, the intestinal flora can in turn metabolize BAs and control their composition and storage in the enterohepatic circulation through an enterohepatic circulation. A number of factors including fasting and ingesting specific nutrients can regulate BA synthesis, intestinal flora composition, and blood circulation hormones to keep systemic metabolic homeostasis and prevent from BA-associated metabolic diseases. Activation of BA receptor signaling offers protection to the gastrointestinal tract against inflammation and damage. Furthermore, various factors, including gene mutations for the BA synthesis and transport, high-fat diets, medications, and circadian rhythm disturbances, are found to mediate the pathologies of multiple diseases that involve cholestatic liver disease, inflammatory bowel disease, diabetes, obesity, tumors, and related metabolic disorders (Li and Chiang, 2014; Fiorucci et al., 2021; Fu et al., 2021; Perino et al., 2021; Yang et al., 2021; Shi et al., 2023). In recent years, several researches have demonstrated that BAs have antitumor properties in various cancer cell types, such as tamoxifen-resistant breast cancer (Luu et al., 2018; Kovács et al., 2019), oral squamous cell carcinoma (Talebian et al., 2020), cholangiocarcinoma (Lee et al., 2022), gastric cancer (Zhang et al., 2022), colon cancer (Kim E. K. et al., 2017), hepatocellular carcinoma (Fan et al., 2023), prostate cancer (Lee et al., 2017), gallbladder cancer (Lin et al., 2020; Li et al., 2022), neuroblastoma (Trah et al., 2020) etc., by inhibiting cancer cell proliferation and migration. In addition, new BA derivatives have been synthesized in several laboratories to investigate their anticancer properties. These derivatives were demonstrated to trigger cell death in cancer cells and exhibit anti-tumor properties (Katona et al., 2009; Sreekanth et al., 2013; Tang et al., 2018; Markov et al., 2019; Agarwal et al., 2021; Melloni et al., 2022). This review discusses the anticancer activity of natural BAs and synthetic derivatives in cancer cells and their signaling pathways and therapeutic approaches potentially targeted to human cancers.

## 2 Bile acid biosynthesis

**FIGURE 1:**
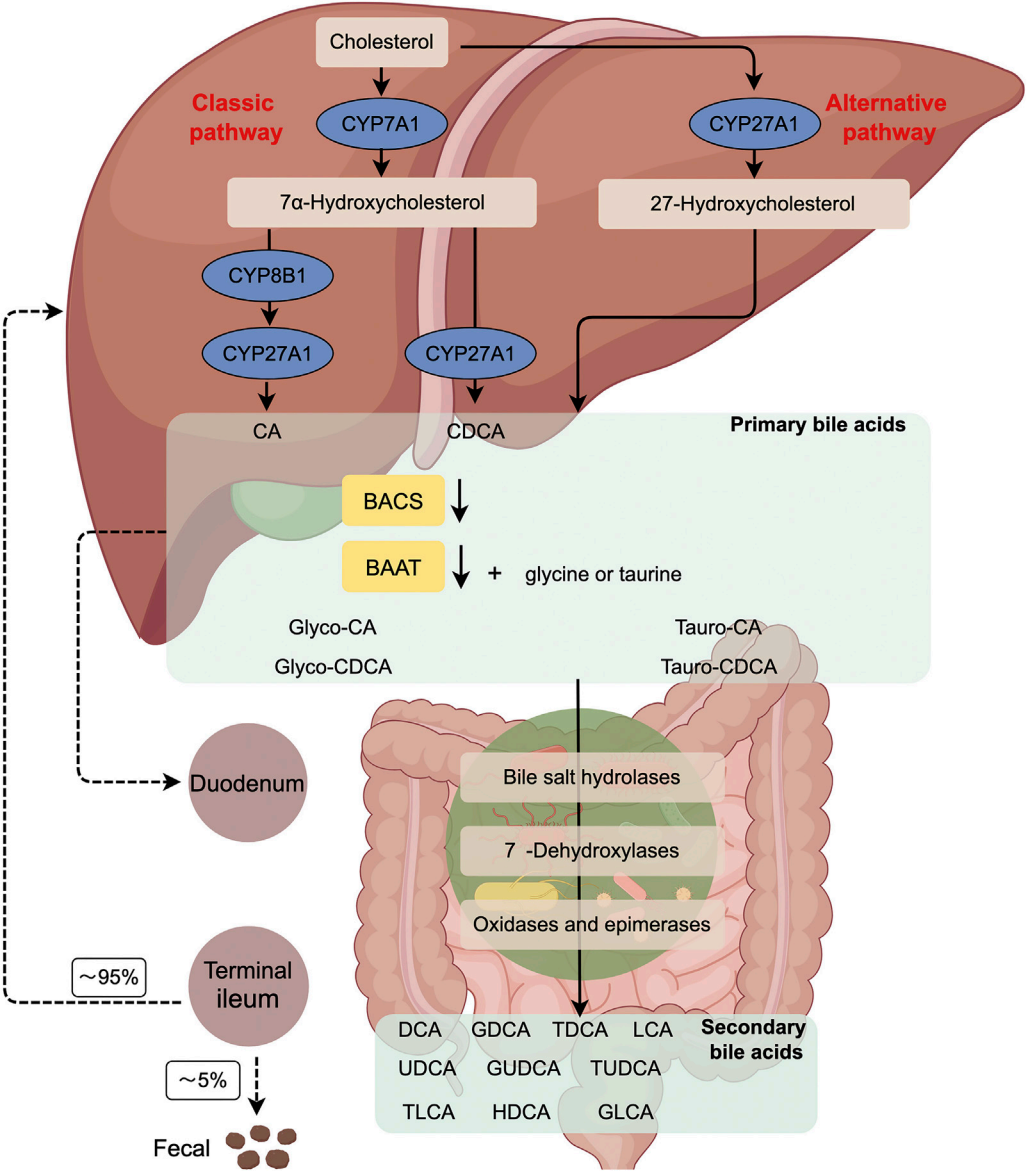
The Diagram of the classical and alternative bile acid synthesis in human. Primary BAs are generated from cholesterol by the classic (CYP7A1-mediated) or alternative (CYP27A1-mediated) pathway. Subsequently, BACS and BAAT catalyze the conjugation of BAs with glycine or taurine in the liver, resulting in the formation of bile salts. Once released into the gut, these bile acids undergo modification by the gut microbiome, leading to the production of secondary BAs. Approximately 95% of the BAs that reach the terminal ileum are reabsorbed, allowing for their recycling by the liver. CYP7A1, cholesterol 7α-hydroxylase; CYP27A1, sterol 27- hydroxylase; BACS, BA-CoA synthetase; BAAT, BA-CoA: amino acid N-acyltransferase; CA, cholic acid; CDCA, chenodeoxycholic acid; CYP8B1, sterol12α-hydroxylase.

BAs are the final products of cholesterol catabolism in the liver and consist of a variety of lipid-soluble acids, including cholic acid (CA), deoxycholic acid (DCA), chenodeoxycholic acid (CDCA), glycochenodeoxycholic acid (GCDCA), ursodeoxycholic acid (UDCA), glycoursodeoxycholic acid (GUDCA), glycodeoxycholic acid (GDCA), glycocholic acid (GCA), taurocholic acid (TCA), taurochenodeoxycholic acid (TCDCA), tauroursodeoxycholic acid (TUDCA), taurodeoxycholic acid (TDCA), lithocholic acid (LCA), glycolithocholic acid (GLCA), and taurolithocholic acid (TLCA) (Li et al., 2022). BAs have two main ways of biosynthesis: classical and alternative synthetic pathways (Figure 1) (Chiang, 2009). The microsomal rate-limiting enzyme cholesterol 7α-hydroxylase (CYP7A1) initiates the classical BA synthesis pathway by which CYP7A1 oxidizes cholesterol into 7α-hydroxycholesterol. Subsequently, 3β-hydroxy∆5-C27-steroid dehydrogenase (HSD3B7) catalyzes the conversion of 7α-hydroxycholesterol to 7α-hydroxy-4-cholesten-3-one (C4), a precursor of the primary BAs, CA and CDCA. C4 also serves as a common serum biomarker used to evaluate levels of BA synthesis. Microsome sterol 12α-hydroxylase (CYP8B1) can convert C4 to 7α, 12α-dihydroxy-4-cholestene 3-1thatisfurther metabolized to be a precursor of the CA 3-alpha, 7-alpha, 12-alpha trihydroxycholestanoicacid (THCA) by aldo-keto reductases (AKR) AKR1D1/1C4 and mitochondrial sterol cholesterol 27-hydroxylase (CYP27A1). In the absence of 12α-hydroxylation, C4 undergoes conversion into 3α, 7α dihydroxycholestanoic acid (DHCA), which serves as the precursor for CDCA. THCA and DHCA are transported to peroxisomes for steroid side chain cleavage, which occurs similarly to fatty acid β-oxidation.

Initially, BA coenzyme A (CoA) synthase (BACS; SLC27A5) catalyzes THCA and DHCA into acyl-CoA thioesters. Subsequently, these thioesters are transported to peroxisomes through the peroxisomal BA-acyl transporter ABCD3. Among them, α-methylacyl-CoA racemase (AMACR), acyl-CoA oxidase (ACOX2), and D-bifunctional enzyme (ACOX2) are the most common enzymes. HSD17B4 completes the racemization, hydration, and dehydration steps. Finally, the sterol carrier protein x (SCPx) cleans releases propanoyl-coA from the steroid side chains of THCA and DHCA to form cholyl-coA and chenodeoxycholyl-coA, respectively. BA-coA: amino acid N-acyltransferase (BAAT) couples cholyl-coA and CDCA-coA to taurine or glycine to form T/G-CA and T/G-CDCA, respectively (Perino et al., 2021).

In the alternative synthetic pathway, CYP27A1 is crucial in converting cholesterol to 27-hydroxycholesterol and 3β-hydroxy-5-cholesterol in the liver, macrophages, and adrenal glands. Oxysterol 7α-hydroxylase (CYP7B1) hydroxylates C7, resulting in the formation of 7α, 27-dihydroxycholesterol and 3β, 7α-dihydroxy-5 cholestenoic acid. In the brain, cholesterol is converted to 24-hydroxycholesterol by the enzyme sterol 24-hydroxylase (CYP46A1), which is then hydroxylated at the 7α position by a specific sterol 7α-hydroxylase (CYP39A1) in the liver. The oxysterols generated in extrahepatic tissues can serve as substrates for synthesizing CDCA and CA.

Negative feedback mechanisms tightly regulate classical and alternative BA synthesis pathways (Di Ciaula et al., 2017; Collins et al., 2023). In human, the synthesis of BAs is primarily derived from the classical pathway, whereas approximately 50% of BAs in rodents are synthesized from the alternative pathway. CA and CDCA are the two primary BAs synthesized in the human liver. CDCA, a hydrophobic BA, undergoes further conversion to α-muricholic acid (α-MCA) by a mouse-specific enzyme sterol-6β-hydroxylase (Cyp2c70). Furthermore, α-MCA can be epimerized to be 7β-epimer, known as β-MCA. Cyp2c70 also hydroxylizes the secondary BA UDCA produced by gut bacteria to β-MCA. α-MCA and β-MCA are the primary BAs produced in rodent liver and are highly water-soluble and non-toxic. In human, bacterial 7β-hydroxysteroid dehydrogenase (7β-HSDH) converts merely 2% of CDCA as a secondary BA to the 7β-epidermoid UDCA that is a highly water-soluble and non-toxic BA.

## 3 The anticancer effect of natural BAs

**FIGURE 2:**
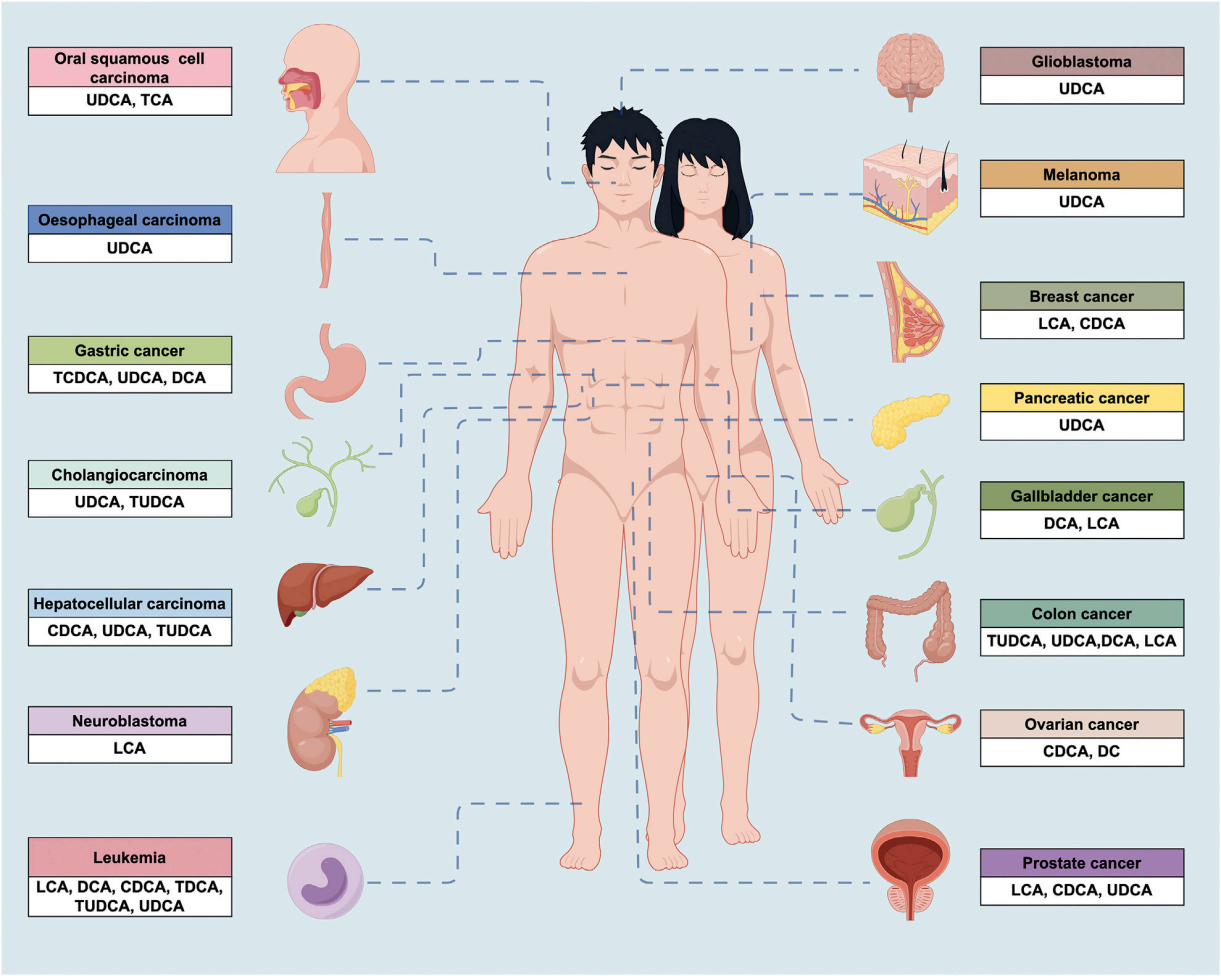
The anticancer properties of BAs in a wide variety of cancers. UDCA, ursodeoxycholic acid; TCA, taurocholic acid; TCDCA, taurochenodeoxycholic acid; DCA, deoxycholic acid; TUDCA, tauroursodeoxycholic acid; CDCA, chenodeoxycholic acid; LCA, lithocholic acid; TDCA, taurodeoxycholic acid.

BAs are typically appreciated as major signal molecules that act as emulsifiers in dietary lipid digestion and absorption (Melloni et al., 2022). Interestingly, they are found to intervene the development of diabetes, metabolic disorders, heart disease, respiratory ailments, and tumors (Collins et al., 2023; Shi et al., 2023). In this section, our attention primarily focuses on exploring the anticancer impacts of natural BAs (Figure 2.) on cancer cells *in vitro* (e.g., proliferation, invasion, migration, and adhesion) (Table 1).

**TABLE 1:** Tumor suppressive effects of natural BAs on cancers.

Cancer types	Cell lines	Bile acids	Phenotype	Effects	Refs
Glioblastoma	A172, LN229	UDCA	Cell viability	Inducing ROS production, arresting the G1 phase, reducing MMP and inducing endoplasmic reticulum stress	Yao et al. (2020)
Neuroblastoma	BE (2)-m17, SK-n-SH, SK-n-MCIXC and Lan- 1	LCA	Cell death	Activating apoptotic pathways	Goldberg et al. (2011)
	WT CLS1, SK NEP1	LCA	Proliferation	Activating apoptotic pathways	Trah et al. (2020)
Oral Squamous Carcinoma	HSC-3	UDCA	Cell viability	Inducing apoptosis via caspase activation	Pang et al. (2015)
Pancreatic cancer	HPAC, Capan1	UDCA	EMT, stem cell formation	Upregulating intracellular ROS and down-regulating *Prx2*	Kim E. K. et al. (2017)
	PANC-1	Bile acids	Proliferation, EMT	Inducing apoptosis via ROS and EMT pathway	Zhu et al. (2022)
	PANC-1, MIA PaCa-2, PGHAM-1	DCA, CA	Proliferation, cytoplasmic microvilli loss and organelles vacuolization	Increasing the percentage of G0+G1 phase cells	Wu et al. (2003)
		GCA, TDCA	Proliferation, cytoplasmic microvilli loss and organelles vacuolization	Elevating the S phase cell number	Wu et al. (2003)
Prostate cancer	DU145	UDCA	Proliferation	Activating apoptotic pathways	Lee et al. (2017)
	LNCaP, PC-3	LCA	Proliferation	Activating apoptotic pathways	Goldberg et al. (2013)
	PC-3, DU145	LCA	Cell viability	Inducing ER stress, autophagy, and mitochondrial dysfunction	Gafar et al. (2016)
	LNCaP, DU145	CDCA	Proliferation	Activating FXR and accumulating lipids via the SREBF pathway	Liu et al. (2016)
	LNCaP	CDCA	Proliferation	Activating FXR and upregulating PTEN.	Liu et al. (2014)
Hepatocellular carcinoma	Huh-BAT, HepG2	UDCA	Proliferation	Activating ERK and dephosphorylating STAT3	Lee et al. (2018)
	B16-F10, MC38, LLC, A549 and SW480	UDCA	Treg cell differentiation and activation	Enhancing antitumor immunity by serving as a TGF-β inhibitor	Shen et al. (2022)
	HepG2	TUDCA	Proliferation and invasion	Suppressing cell death and inflammation mediated by ER stress	Vandewynckel et al. (2015)
	HepG2, BEL7402	UDCA	Proliferation	Blocking the cell cycle and regulating the expression of *Bax/Bcl-2* genes	Liu et al. (2007), Liu et al. (2015)
	HepG2	UDCA	Proliferation	Inducing apoptosis via regulation of the expressions of *Smac* and *Livin* and caspase 3	Zhu et al. (2014)
	Huh-Bat, SNU761, SNU475	UDCA	Proliferation	Inhibiting proteasomal DLC1 degradation	Chung et al. (2011)
	HepG2, SK-Hep1, SNU-423, Hep3B	UDCA	Proliferation	Inhibiting ROS production and activating the p53-caspase 8 pathway	Lim et al. (2010)
	HepG2, Huh7, mouse hepatoma Hepa 1–6	CDCA	/	Inducing NDRG2 expression through FXR receptor	Langhi et al. (2013)
Gastric cancer	MKN-74	UDCA	Invasion	Suppressing chenodeoxycholic acid-induced PGE2 production	Wu et al. (2018)
	SNU601, SNU638	UDCA	Proliferation	Inducing apoptosisthrough the expression and activation of DR5	Lim et al. (2011)
	SGC-7901	TUDCA	Proliferation, invasion	Inducing Apoptosis	Zhang et al. (2022)
	SCM1	DCA	Cell viability	Causing Ca (2+)-independent apoptosis	Chien et al. (2015)
	SGC-7901	DCA	Proliferation	Inducing apoptosis through the mitochondrial-dependent pathway	Song et al. (2013)
	SNU601	UDCA	Proliferation	Inducing apoptosis via MEK (MAPK)/ERK pathway	Lim et al. (2012)
	SNU601	UDCA	Proliferation	Inducing apoptosis via CD95/Fas death receptor, downregulating ATG5 level and preventing autophagic pathway	Lim and Han (2015)
	BGC-823	DCA	Proliferation	Activating p53 mediated pathway	Yang et al. (2015)
	SNU-216, MKN45	DCA	Invasion, migration	Inducing *MUC2* expression	Pyo et al. (2015)
Oesophageal cancer	SKGT-4, OE33	UDCA	/	Inhibiting NF-κB, AP-1 activation and *COX-2* upregulation	Abdel-Latif et al. (2016)
Colon cancer	HCT116	UDCA	Apoptosis	Modulating EGFR/Raf-1/ERK signaling	Im and Martinez (2004)
	HCT116	UDCA	Apoptosis	Mediating the PI3K, MAPK, or cAMP pathways	Saeki et al. (2012)
	HCT116	UDCA	Proliferation	Inhibiting the expression of c-Myc and cell cycle regulatory molecules	Peiró-Jordán et al. (2012)
	HT29, HCT116	UDCA	Proliferation	Regulating ROS production, activating ERK1/2	Kim Y. J. et al. (2017)
	HCT116	UDCA	Progression	Inhibiting interleukin β1 and blocking NF-κB and AP-1 activation	Shah et al. (2006)
	HT-29	UDCA	Proliferation	Promoting endocytosis and degradation of EGFR receptor	Feldman and Martinez (2009)
	HCT116, COLO 205	TUDCA	Progression	Suppressing NF-κB signaling	Kim et al. (2019)
	Caco-2, HT29C19A	LCA	Anti-inflammatory signals	Blocking inflammatory signals	Sun et al. (2008)
	HCT116	LCA	Proliferation	Activating p53 and binding to MDM4 and MDM2	Vogel et al. (2012)
	HCT116	DCA, CDCA	Proliferation	Induce apoptosis	Powell et al. (2001)
	HCT116	DCA	Proliferation	Inducing apoptosis via AP-1 and C/EBP mediated GADD153 expression	Qiao et al. (2002)
	HCT116	DCA	Proliferation	Inhibiting cell growth and inducing apoptosis physiologically	Zeng et al. (2015)
Cholangiocarcinoma	Mz-ChA-1	TUDCA	Proliferation	Involving in MAPK p42/44 and PKCα	Alpini et al. (2004)
Gallbladder cancer	NOZ, GBC-SD, EH-GB1	DCA	Proliferation	Interfering with miR-92b-3p maturation	Lin et al. (2020)
	NOZ, EH-GB1	LCA	Proliferation	Downregulating GLS-mediated glutamine metabolism and inducing ferroptosis	Li et al. (2022)
Breast cancer	MCF7, MDA-MB-231	LCA	Proliferation	Inducing *TGR5* expression, inhibiting lipogenesis and reducing *ERα* expression	Luu et al. (2018)
	MCF7, 4T1	LCA	Proliferation, EMT, VEGF production, immune response	Activating TGR5 receptor	Mikó et al. (2018)
	MCF7, 4T1	LCA	Oxidative stress	Inducing NRF2/NFE2L2 dependent oxidative/nitrosative stress	Kovács et al. (2019)
	MCF7	CDCA	Tamoxifen-resistance	Activating FXR receptor	Giordano et al. (2011)
	MCF7, MDA-MB-231	CDCA	Cell death	Activating FXR receptor	Alasmael et al. (2016)
Ovarian cancer	OVCAR3	CDCA, DCA	Proliferation	Upregulating *BRCA1* and downregulating *ESR1* gene expression	Jin et al. (2018)
	A2780	CDCA, DCA	Proliferation	Inducing apoptosis	Horowitz et al. (2007)
Leukemia	T leukemia cell line	UDCA, TUDCA	Proliferation	Delaying cell cycle progression	Fimognari et al. (2009)
	THP1, Molm-13	CDCA	Proliferation, inhibition of M2 macrophage polarization	Accumulating LDs and lipid peroxidation via ROS/p38 MAPK/DGAT1 pathway	Liu et al. (2022)
	HL60, THP-1	DCA, CDCA, LCA	Proliferation and differentiation	Accumulating the G0/G1 transition and inhibiting the differentiation	Zimber et al. (1994)
Melanoma	M14, A375	UDCA	Proliferation	Inducing ROS-triggered mitochondrial-associated pathway	Yu et al. (2019)

*GCA*, glycocholic acid; *TDCA*, *t*aurodeoxycholic acid; *UDCA*, ursodeoxycholic acid; *TUDCA*, tauroursodeoxycholic acid; *ROS*, reactive oxygen species; *LCA*, lithocholic acid; *EMT*, epithelial–mesenchymal transition; *DCA*, deoxycholic acid; *CA*, cholic acid; *CDCA*, chenodeoxycholic acid; *Prx2*, peroxiredoxin II; *FXR*, farnesoid X receptor; *Dlc1*, deleted in Liver Cancer 1; *AP-1*, activator protein-1; *COX2*, cyclooxygenase-2; *Bax*, Bcl-2-associated X protein; *Bcl-2*, B-cell lymphoma 2; *PGE2*, prostaglandin E2; *MDM2*, mouse double minute 2; *MDM4*, double Minute 4; *c-Myc*, myc-Related translation/localization regulatory factor; *ERK*, extracellular signal-regulated kinase; *MAPK*, mitogen-activated protein kinase; *NF-κB*, nuclear factor κappa-light-chain-enhancer of activated B cells; *PI3K*, phosphatidylinositol 3-kinase; *STAT3*, signal transducer and activator of transcription 3; *TGR5*, G protein-coupled bile acid receptor 5; *VEGF*, vascular endothelial growth factor; *DR5*, death receptor 5; *NDRG2*, N-Myc downstream regulated gene 2; *SREBF*, sterol regulatory element-binding factor; *MUC2*, mucin 2; *ATG5*, Autophagy Related 5; *cAMP*, cyclic adenosine monophosphate; *GADD153*, growth arrest- and DNA, damage-inducible gene 153; *BRCA1*, breast cancer type 1 susceptibility protein; *PTEN*, phosphatase and tensin homolog; *EGFR*, epithelial growth factor receptor; *C/EBP*, CCAAT/enhancer-binding protein beta; *RAF1*, Raf-1, Proto-Oncogene; *NRF2*, nuclear factor erythroid 2-related factor 2; *LDs*, lipid droplets; *PKCα*, protein kinase C α; *MMP*, mitochondrial membrane potential; *GLS*, glutaminase; *TGF-β*, Transforming growth factor-β; *Smac*, second mitochondria-derived activator of caspase; *ESR1*, Estrogen Receptor 1.

### 3.1 Glioblastoma (GB)

Glioblastoma (GB) is the most prevalent and aggressive form of adult human brain tumor. Despite the implementation of aggressive regimens involving surgery, radiation and chemotherapy, the prognosis for GBM patients remains poor with a median survival of 15 months (Schaff and Mellinghoff, 2023). UDCA demonstrates the ability to penetrate through the blood-brain barrier; thus it implicates powerful activity to block brain tumor (Palmela et al., 2015). Yao et al. (2020) demonstrated that UDCA inhibited GB progression in multiple aspects such as inducing G1 phase arrest, reducing mitochondrial membrane potential (MMP), promoting overproduction of reactive oxygen species (ROS), and inducing endoplasmic reticulum (ER) stress. Combining UDCA with bortezomib (BTZ) also synergistically enhances the PERK/ATF4/CHOP pathway and protracts ER stress (Yao et al., 2020).

### 3.2 Neuroblastoma (NB)

Nephroblastoma (NB) ranks as the second most common intraabdominal cancer and the fifth most prevalent malignancy in children (Walz et al., 2023). Extensive research efforts have enhanced the survival rate from less than 30% to high 85%–90%. Nevertheless, the relapse rate persists within the range of 15%–50% (Saltzman et al., 2023). Strikingly, LCA effectively induced NB cell death *in vitro* through apoptosis without neuron cytotoxicity. This elimination was achieved by triggering the intrinsic (initiator caspase-9 activation) and extrinsic apoptosis pathways (the initiator caspase-8 activation) (Goldberg et al., 2011; Trah et al., 2020).

### 3.3 Oral squamous cell carcinoma (OSCC)

Oral cancers represent prevalent malignant tumors within the head and neck and are primarily classified as squamous cell carcinomas that involve the transformation of mucous membranes in the gums, tongue, and face into cancerous tissues (Tan et al., 2023). UDCA has demonstrated potential in preventing gum and periodontal dysfunctions, as well as reducing gum bleeding (Pang et al., 2015). As the result, it is suggested that UDCA may hold promise in the treatment of oral cancers. Pang et al. (2015) demonstrated that UDCA triggered apoptosis in oral squamous cell cancer cells (HSC-3) via caspase activation. They also found that high UDCA levels exhibited cytotoxic effects *in vitro* (Pang et al., 2015).

Elevated levels of BAs have been recently known to be associated with impaired immune cell function, increased patient morbidity and even mortality. Consequently, high levels of BAs are considered immune suppressors, in which TCA is the most potent one of tumor immune inhibitors (Liu et al., 2018). Talebian et al. (2020) reported that TCA exhibited anti-inflammatory activities in human OSCC cells *in vitro*.

### 3.4 Oesophageal carcinoma

Esophageal carcinoma is prevalent in the developing countries and is characterized with significantly high morbidity and mortality, whereas its incidence is declining in the developed countries (Li et al., 2023). Abdel-Latif et al. (2016) revealed that pretreatment with UDCA effectively inhibited DCA-induced nuclear factor kappa B (NF-κB) and activator protein-1 (AP-1) DNA-binding activities in oesophageal carcinoma cells, thus decreasing cell survival.

### 3.5 Cholangiocarcinoma

Cholangiocarcinoma represents a malignant tumor associated with 20%–30% rate of 5-year survival even after resection. For those unable to undergo resection, the prognosis is especially poorer in which most patients fail to survive longer than 2 years (Greten et al., 2023). Although non-surgical palliative chemotherapy and radiation therapy are alternatively optional, their outcomes have not yielded satisfactory results. UDCA inhibited the growth of cholangiocarcinoma, and the combined UDCA and gefitinib displayed a more robust effect. Thus, UDCA demonstrates a potential adjuvant or palliative anticancer drug, providing a therapeutic option to enhance the effects of other chemotherapeutic agents synergistically (Lee et al., 2022). UDCA suppressed cholangiocarcinoma cell proliferation and invasiveness by triggering apoptosis, activating p53, and blocking DCA-induced activated EGFR-ERK and PI3K-AKT signaling (Lee et al., 2021). TUDCA impeded the proliferation of bile duct cancer cells by activating the mitogen-activated protein kinase (MAPK) p42/44 and PKCα signaling pathways (Alpini et al., 2004).

### 3.6 Gallbladder cancer (GBC)

Gallbladder cancer is a highly malignant disease that is often misdiagnosed at early stages. Thus, rapid development of GBC at later stages has largely limited the possibility of surgical intervention, leading to a poor prognosis (Li et al., 2014; Song et al., 2020a; Geng et al., 2022; Wang et al., 2023a; Wang et al., 2023b). DCA treatment has been found to halt GBC cell proliferation and reduce miR-92b-3p expression in an m^6^A-dependent post-transcriptional modification manner by facilitating METTL3 dissociation from METTL3-METTL14-WTAP complex and thus inactivating PI3K/AKT signaling pathway (Lin et al., 2020). LCA treatment has demonstrated tumor-suppressive function in GBC by decreasing glutaminase expression, interfering with glutamine metabolism and reducing GSH/GSSG and NADPH/NADP^+^ ratios. These effects lead to cellular ferroptosis and suppress tumor growth of GBC cell lines (Li et al., 2022).

### 3.7 Hepatocellular carcinoma (HCC)

Hepatocellular carcinoma (HCC) accounts for 85%–95% of primary liver cancer. Approximately 80% of HCC patients are diagnosed at advanced stages when surgical intervention is not applicable. The overall 5-year survival rate is less than 30% in advanced HCC patients as most of those patients with 80% experience cancer recurrence (Brown et al., 2023). Consequently, there is an urgent need to elucidate the mechanisms underlying HCC progression and develop effective therapy. CDCA robustly induced the expression of N-Myc downstream-regulated gene 2 (NDRG2) to hinder the proliferation of hepatoma cells (Langhi et al., 2013). Combining UDCA with anti-PD-1 enhanced anticancer immunity and promoted the development of tumor-specific immune memory. Additionally, UDCA phosphorylated transforming growth factor-beta (TGF-β) at the T282 site by activating the TGR5-cAMP-PKA axis, which increased the binding of TGF-β to the carboxyl terminus of the Hsc70-interacting protein. Combination therapy using anti-PD-1 or anti-PD-L1 antibody together with UDCA was more effective in treating tumor patients than singleanti-PD-1 or anti-PD-L1 antibody (Shen et al., 2022). Combining sorafenib and UDCA chemotherapy showed efficacy in advanced HCC by inhibiting cell proliferation and inducing apoptosis through ROS-dependent activation of ERK and Stat3 dephosphorylation (Lee et al., 2018). TUDCA attenuated apoptosis induced by ER stress (Vandewynckel et al., 2015). UDCA suppressed HCC growth *in vivo* in a dose- and time-dependent apoptosis fashion by upregulating the Bax to Bcl-2 ratio, *Smac*, *Livin* and caspase-3 expressions (Zhu et al., 2014; Liu et al., 2015), serving as a therapeutic candidate for HCC treatment. UDCA also exhibited selective ability to inhibit proliferation and induce apoptosis in HCC cell lines by disrupting the cell cycle and modulating the expression of Bax/Bcl-2 genes (Liu et al., 2007). Likewise, UDCA acted as an anti-proliferative agent in HCC by inducing DLC1 protein expression and inhibiting proteasomal DLC1 degradation (Chung et al., 2011). In HepG2 cells, UDCA transformed oxaliplatin-induced necrosis into apoptosis by inhibiting ROS generation and activating the p53-caspase 8 pathway. The combination of UDCA with chemotherapy effectively inhibited HCC by diminishing inflammatory responses (Lim et al., 2010).

### 3.8 Pancreatic cancer

Pancreatic cancer shows a notably low survival rate primarily owe to late diagnosis and resistance to therapies (Halbrook et al., 2023). The adverse effects of these chemotherapy treatments are also detrimental. Thus, optimal treatment remains to be developed. UDCA displayed the ability to prevent epithelial-mesenchymal transition (EMT) in pancreatic cancer cell lines, indicating its potential as an agent with antineoplastic properties (Kim Y. J. et al., 2017). UDCA suppressed intracellular ROS and Prx2 levels, EMT and stem cell formation in pancreatic cancer cells. These findings suggest that UDCA’s antioxidant effects may provide favorable therapeutic benefits for patients with pancreatic cancer (Kim Y. J. et al., 2017). A high BA level could inhibit cell proliferation and migration by inducing ROS and EMT pathways, thereby promoting apoptosis of pancreatic cancer cells (Zhu et al., 2022). BAs could reduce the proliferation of pancreatic cancer cells due to direct cytotoxicity (Wu et al., 2003). Specifically, DCA and CA induced cell cycle arrest, while GCA and TDCA elevated the S phase cell number, suggesting enhanced DNA synthesis and progression through the cell cycle (Wu et al., 2003).

### 3.9 Gastric cancer (GC)

Gastric cancer (GC) is one of the leading causes of cancer-related mortality worldwide. Most patients are diagnosed at advanced stages due to the neglect of minimal symptoms at earlier stages and the lack of regular early screening. Systemic therapies for GC including chemotherapy, targeted therapy, and immunotherapy, have been notably practiced in recent years (Guan et al., 2023). However, the favorable efficacy remains to be evaluated. TCDCA inhibited gastric cancer proliferation and invasion and induced apoptosis. Traditional Chinese medicine in experimental studies offered encouraging evidence for the potential application in the blockade of tumor (Zhang et al., 2022). DCA triggered apoptosis in gastric carcinoma cells by activating intrinsic mitochondrial-dependent, p53-mediatedcell death pathway (Yang et al., 2015). Furthermore, the upregulation of the Bax/Bcl-2 ratio and disruption of the mitochondrial membrane potential significantly contributed to the induction of DCA-mediated apoptosis in gastric carcinoma cells (Song et al., 2013). DCA also induced MUC2 expression in GC cells, inhibiting tumor progression. Accordingly, MUC2-expressing GC cells demonstrated decreased Snail expression (Pyo et al., 2015). UDCA drove apoptosis and autophagy, overcoming drug resistance (Lim and Han, 2015). Additionally, UDCA and DCA demonstrated suppressive effects in gastric cancer cells by activating the ERK signaling molecules (Lim et al., 2012). UDCA inhibited invasion by suppressing chenodeoxycholic acid induced PGE2 production (Wu et al., 2018). Furthermore, UDCA promoted GC apoptosis by activating the death receptor 5 (DR5) in lipid rafts (Lim et al., 2011).

### 3.10 Colon cancer

Colon cancer represents approximately 10% of all human cancers worldwide and, is also a leading cause of cancer-related deaths (Gallois et al., 2023). Except the essential early diagnosis and prevention required for clinic practice, effective therapies emerge as the most powerful aspect to improve patient survival. BAs play a causal role in colon cancer by inducing DNA damage (Kandell and Bernstein, 1991). TUDCA inhibited the NF-κB signaling pathway and alleviated colitis-associated tumorigenesis, indicating the valuable therapeutic means for colon cancer treatment (Kim et al., 2019). DCA increased intracellular ROS, genomic DNA breakage, and expression of ERK1/2, caspase 3, and PARP. In addition, DCA inhibited colonic cell proliferation through activation in the cell cycle and apoptosis pathways (Zeng et al., 2015). DCA exerted common and distinct effects on cell cycle, apoptosis, and MAP kinase pathway in human colon cancer cells (Zeng et al., 2009). DCA inhibited the proliferation by inducing apoptosis through AP-1 and C/EBP-mediated GADD153 expression (Qiao et al., 2002). Both DCA and CDCA suppressed cell proliferation by inducing apoptosis (Powell et al., 2001). UDCA suppressed cell proliferation by regulating oxidative stress in colon cancer cells (Kim E. K. et al., 2017). Treatment of colon carcinoma cells with UDCA inhibited cell proliferation by suppressing c-Myc expression and several cell cycle regulatory molecules (Peiró-Jordán et al., 2012). UDCA suppressed cell growth by inhibiting the mitogenic activity of receptor tyrosine kinases such as EGFR through increased receptor degradation (Feldman and Martinez, 2009). UDCA exerted a partial inhibitory effect on DCA-induced apoptosis via disrupting EGFR/Raf-1/ERK signaling pathway (Im and Martinez, 2004). UDCA prevented colon tumor and polyp formation by balancing the toxic effects of DCA and enhanced the potential cytoprotective effects of muricholic acids in the water-soluble fraction in rat feces (Batta et al., 1998). UDCA induced apoptosis by blocking the PI3K, MAPK, or cAMP pathways (Saeki et al., 2012). UDCA inhibited interleukin β1 and blocking NF-κB and AP-1 activation in colon cells (Shah et al., 2006). TUDCA augmented the cytotoxicity of hydrophobic BAs *in vitro*, and gaining a better understanding of how BAs interact in the colon can significantly impact the alteration of tumor promotion (Shekels et al., 1996). LCA was found to activate the vitamin D receptor (VDR), blocking inflammatory signals in colon cells (Sun et al., 2008). LCA also activated p53 that binds to MDM4 and MDM2, abrogating cell proliferation (Vogel et al., 2012).

### 3.11 Breast cancer

Breast cancer continues to be the first ranked cancer in women, which is characterized by significant disease heterogeneity, metastasis, and therapeutic resistance (Nolan et al., 2023). Growing evidence has found that LCA blocked breast cancer cell proliferation by stimulating oxidative stress that is under mined during breast cancer progresses (Kovács et al., 2019). LCA was able to regulate lipid metabolism reprogramming to inhibit breast cancer cells (Luu et al., 2018). Moreover, natural BAs negatively impacted on human breast cancer cell growth and steroid receptor function (Baker et al., 1992). Like LCA in breast cancer treatment, CDCA prompted cell death and resensitized tamoxifen resistant breast cancer (Giordano et al., 2011; Alasmael et al., 2016). Additionally, LCA exerted inhibitory effects on breast cancer proliferation, epithelial-mesenchymal transition (EMT), vascular endothelial growth factor (VEGF) production, and immune responses through the activation of the Takeda-G-protein-receptor-5 (TGR5) receptor (Mikó et al., 2018).

### 3.12 Prostate cancer

In man, prostate cancer is ranked as the most widespread cancer globally and is the second leading cause of cancer-related mortality in most developed countries. It is of note that a significant population of elderly patients are unable to withstand the conventional chemotherapy (Hamdy et al., 2023). In addition, increasing resistance to hormonal therapy has emerged as the substantial challenge in clinical treatment. Hence, alternative new drug development has been largely taken into account. LCA exhibited potent and non-selective effects on prostate cancer cells while sparing highly differentiated podocytes at lower concentrations, rendering it potential for an effective anticancer drug (Trah et al., 2020). LCA induced approximately 98% of cancer cell cytotoxicity at nominal concentrations in cultured medium (Goldberg et al., 2013). LCA induced autophagy and ER stress in PC-3 cells. However, this signature was found to be associated with initial protection and subsequent consequences rather than the ultimate cytotoxicity and mitochondrial dysfunction mediated by ROS (Gafar et al., 2016). LCA suppressed the proliferation of androgen-dependent (AD) LNCaP prostate cancer cells by inducing an apoptotic pathway (partially dependent on caspase-8 activation). Notably, LCA increased Bid and Bax cleavage, Bcl-2 downregulation, mitochondrial outer membrane permeabilization, and caspase-9 activation. UDCA drove apoptosis in prostate cancer cells by activating extrinsic and intrinsic apoptotic pathways (Lee et al., 2017). CDCA and DCA were shown to destabilize HIF-1α, significantly suppressing clonogenic growth, invasion, and migration (Liu et al., 2016). CDCA inhibited prostate cancer cells via activating the Farnesoid X receptor (FXR) and upregulating phosphatase and tensin homolog (PTEN) (Liu et al., 2014).

### 3.13 Ovarian cancer

Ovarian cancer is an aggressive disease that is often detected at advanced stages and typically exhibits a strong initial response to platinum-based chemotherapy. Despite this, the majority of patients experience relapse after the initial surgery and chemotherapy, implicating the critical necessity for the development of new therapeutic strategies (Konstantinopoulos and Matulonis, 2023). CDCA and DCA exhibited noteworthy cytotoxic activity in ovarian cancer cells by inducing apoptosis (Horowitz et al., 2007). CDCA and DCA could upregulate BRCA1 and downregulate ESR1 expression to inhibit BRCA1 mutated ovarian cancer progression (Jin et al., 2018).

### 3.14 Leukemia

Leukemia represents a highly fatal hematologic malignancy characterized by the accumulation of poorly differentiated myeloid cells in the bone marrow and blood, even in other tissues and organs. This widespread feature results ultimately in systemic dysfunction (DiNardo et al., 2023). To date, numerous research endeavors have been added to enhance treatment outcomes (Kayser and Levis, 2023), yet the rate of complete remission remains low. CDCA suppressed acute myeloid leukemia (AML) progression by promoting both lipid droplets (LD) accumulation and lipid peroxidation via ROS/p38 MAPK/DGAT1 pathway. CDCA also inhibited the polarization of M2 macrophages, contributing to its anti-leukemic properties (Liu et al., 2022). DCA, UDCA, TDCA, and TUDCA induced a delay in cell cycle progression in the human T leukemia cell line. Furthermore, DCA significantly increased the apoptotic cell fraction. DCA, CDCA and LCA inhibited the proliferation by accumulating the G0/G1 transition and inhibiting the differentiation (Zimber et al., 1994). Given the hydrophobic properties of DCA accounted for its cytotoxicity, it is possible to develop its derivatives as new anti-leukemia drugs for cancer therapy (Fimognari et al., 2009).

### 3.15 Melanoma

Melanoma has demonstrated the most lethal form of skin cancer and its incidence within the population has steadily risen in recent years. The high mortality rate of melanoma patients has continued to stimulate new research efforts to the regimens and drug development, expectedly improving the efficacy (Carvajal et al., 2023). UDCA could effectively inhibit melanoma cell proliferation in a time- and dose-dependent manner through cell cycle arrest in the G2/M phase, and cell apoptosis via the ROS-triggered mitochondrial-associated pathway (Yu et al., 2019).

## 4 Synthetic BA derivatives against cancer

Over past recent years, a large volume of researchers have paid the particular attention on modifying the structure of BAs and synthesizing derivatives in order to create novel agents to block cancers. This section mainly focuses on several synthetic derivatives of BAs that have been increasingly reported to inhibit cancer progression effectively (Table 2).

**TABLE 2:** Molecular targets of synthetic bile acid derivatives against cancers.

Bile acids	Derivatives	Cancer type	Cell line	Phenotype	Mechanism	Effects	Refs.
UDCA	HS-1030	Colon cancers	HT-29	Proliferation	Apoptosis	G1 phase arrest, sub-G1-fraction, cyclin D1, E and A and Cdk2, 4, and 6 decrease, Cdk inhibitor, p21WAF1/CIP1 increase	Park et al. (2004)
	HS-1183	Leukemia	Jurkat T cells	Proliferation	Apoptosis	Caspase-3 and -8 down-regulation, PARP cleavage, DNA fragmentation	Choi et al. (2001)
		Colon cancer	HT-29	Proliferation	Apoptosis	Mentioned above	Park et al. (2004)
		Cervical cancer	SiHa	Cell viability	Apoptosis	DNA fragmentation, Bax up-regulation, poly (ADP-ribose) polymerase cleavage	Im et al. (2005)
		Breast cancer	MCF-7, MDA-MB-231	Proliferation	Apoptosis	Apoptotic nuclear changes, sub-G1 population increase, DNA fragmentation	Im et al. (2001)
		Prostate cancer	PC-3	Proliferation	Apoptosis	DNA fragmentation, chromatin condensation, PARP cleavage, cell cycle arrest	Choi et al. (2003)
	UDC-PTX	Leukemia	HL60, NB4	Cell viability	Apoptosis	/	Melloni et al. (2022)
		Colon cancer	RKO, HCT116	Cell viability	Apoptosis	/	Melloni et al. (2022)
CDCA	HS-1199	Leukemia	Mentioned above				Choi et al. (2001)
		Colon cancer	Mentioned above				Park et al. (2004)
		Cervical cancer	Mentioned above				Im et al. (2005)
		Breast cancer	Mentioned above				Im et al. (2001)
		Prostate cancer	Mentioned above				Choi et al. (2003)
		Gastric cancer	SNU-1	Cell viability	Apoptosis	Mitochondrial changes, caspase-3 activation, DNA fragmentation, nuclear condensation	Moon et al. (2004)
		Glioblastoma	U-118MG, U-87MG, T98G, U-373MG	Proliferation	Apoptosis	Mitochondria, caspases and proteasomes	Yee et al. (2005)
	HS-1200	Leukemia	Mentioned above				Choi et al. (2001)
		Colon cancer	Mentioned above				Park et al. (2004)
		Cervical cancer	Mentioned above				Im et al. (2005)
		Breast cancer	MCF-7, MDA-MB-231	Proliferation	Apoptosis	p53 independent pathway activation	Im et al. (2001), Yee et al. (2007)
		Prostate cancer	Mentioned above				Choi et al. (2003)
		Gastric cancer	SNU-1	Cell viability	Apoptosis	Caspase- and mitochondria-dependent fashions	Moon et al. (2004), Jeong et al. (2003)
		Glioblastoma	Mentioned above				Yee et al. (2005)
		Hepatocellular carcinoma	HepG2, Hep3B	Proliferation	Apoptosis	Egr-1 regulation	Liu et al. (2008), Park S. E. et al. (2008)
		Thyroid carcinoma	KAT-18	Cell viability	Apoptosis	Procaspase-3, procaspase-7, and poly (ADP)- ribose polymerase degradation, histone hyperacetylation induction, peripheral chromatin condensation, translocation of apoptosis-inducing factor and caspase-activated DNase decrease	Kim et al. (2009)
	Compound IIIb	Multiple myeloma	KMS-11	Cell viability	Apoptosis	Mcl-1 and PARP-1 cleavage, NF-kB signaling inhibition, DNA fragmentation	El Kihel et al. (2008)
		Glioblastoma multiforme	GBM				
		Colonic carcinoma	HCT-116				
	ent-CDCA	Colon cancer	HT-29 and HCT-116	Proliferation	Apoptosis	CD95 activation, ROS generation, procaspase-8 cleavage	Katona et al. (2009)
	CDC-PTX	Leukemia	Mentioned above				Melloni et al. (2022)
		Colon cancer	Mentioned above				Melloni et al. (2022)
DCA	ent-DCA	Colon cancer	Mentioned above				Katona et al. (2009)
	Compound 9	Duodenal carcinoma	HuTu-80	Cell viability	Apoptosis	ROS-dependent cell death	Markov et al. (2019)
		Hepatocellular carcinoma	HepG2				
		Lung cancer	A549				
		Cervical cancer	KB-3-1				
	4b, 4e, 4d	Lung cancer	A549	Cell viability	Apoptosis	/	Patel et al. (2022)
		Cervical cancer	SiHa				
	6g, 4e	Human osteosarcoma	HOS-CRL-1543	Cell viability	/	/	Agarwal et al. (2018)
	5-FU@Mic-Hyd	Skin cancer	L929, A375	Cell viability	/	/	Pourmanouchehri et al. (2022)
	HD	Squamous cell carcinoma	SCC7	Proliferation		Cytostatic and antiangiogenetic	Park K. et al. (2008)
		Melanoma	B16F10				
LCA	ent-LCA	Colon cancer	Mentioned above				Katona et al. (2009)
	LCA-PIP1	Colon cancer	HCT-116, DLD-1, HCT-8	Cell viability	Apoptosis	/	Singh et al. (2015)
	FHL	Nasopharyngeal carcinoma	KB	Cell viability	Apoptosis	Vessel density decrease	Yu et al. (2007)
	LCA acetate	Hepatoblastoma	HepG2	Proliferation		Binding to VDR	Adachi et al. (2005)
		Colon cancer	SW480	Proliferation		Binding to VDR	
		Leukemia	THP-1	Monocytic differentiation		Binding to VDR	
Bile-acid- appended triazolyl aryl ketones	6af and 6cf	Breast cancer	MCF-7	Cell viability		/	Agarwal et al. (2021)
CA	CA-Tam3- Am	Breast cancer	4T1, MCF-7, T47D and MDA-MB-231	Cell viability	Apoptosis	Molecular charge and hydrophobicity	Sreekanth et al. (2013)
	LLC-202	Liver cancer	HL7702	Proliferation	/	/	Jiang et al. (2023)
	6a, 6c, 6m	Breast cancer	MDAMB231	Cell viability	/	/	Agarwal et al. (2016)
		Colon cancer	HT29				
	6e, 6i, 6m	Glioblastoma	U87				
Piperazinyl bile acid derivative	7b	Multiple myeloma	GBM, KMS-11	Cell viability	Apoptosis	Nuclear and DNA fragmentation	Brossard et al. (2010)
		Colon cancer	HCT-116				
Cationic bile acid-based facial amphiphiles featuring trimethyl ammonium head groups	LCA- TMA1, CDCA- TMA2, DCA- TMA2, and CA-TMA3	Colon cancer	HCT-116 or DLD-1	Proliferation	Apoptosis	Governing membrane interactions, translocation	Singh et al. (2013)
Bile acids	C-7	Breast cancer	MCF-7, MDA-MB-231	Cell viability		/	Bjedov et al. (2017)
		Pancreatic cancer	PC3				
		Ovarian cancer	HeLa				
		Colon cancer	HT-29				
	Compound 27	Prostate cancer	PC3M	Proliferation	Cell cycle	G1 arrest	Mao et al. (2016)
		Colon cancer	HT-29				
		Ovarian cancer	ES-2				
Cholic, ursodeoxycholic, chenodeoxycholic, deoxycholic and lithocholic acids	Piperazinyl bile carboxamide	Colon cancer	DLD-1, HCT-116, and HT-29	Proliferation		/	Brossard et al. (2014)

*HS-1030*, *and HS-1183*, ursodeoxycholic acid derivatives; *HS-1199*, *and HS-1200*, chenodeoxycholic acid derivatives; *ROS*, reactive oxygen species; *CA*, cholic acid; *CA-TMA3*, cholic acid based amphiphile; *CA-Tam3-Am*, cholic acid−tamoxifen conjugate; *CDCA*, chenodeoxycholic acid; *CDC-PTX*, chenodeoxycholic-paclitaxel hybrid; *CDCA-TMA2*, chenodeoxycholic acid based amphiphiles; *LCA*, lithocholic acid; *LCA-PIP1*, lithocholic acid–piperidine 1; *LCA-TMA1*, lithocholic acid based amphiphile; *norUDCA*, nor-ursodeoxycholic acid; *UDCA*, ursodeoxycholic acid; *UDC-PTX*, ursodeoxycholic-paclitaxel hybrid; *ent-CDCA*, enantiomers of chenodeoxycholic acid; *ent-DCA*, enantiomers of deoxycholic acid; *ent-LCA*, enantiomers of lithocholic acid; *6af and 6cf*, bile acid-added triazolyl aryl ketones; *7b*, piperazinyl bile acid derivative; *DCA*, deoxycholic acid; *DCA-TMA2*, deoxycholic acid based amphiphiles; *PARP*, poly (ADP-ribose) polymerase; *Mcl-1*, myeloid leukemia 1; *compound 9*, chenodeoxycholic acid derivative; *compound IIIb*, chenodeoxycholic acid-substituted piperazine conjugate; *VDR*, vitamin D receptor; *Egr-1*, early growth response-1; *p-ULK1*, phosphorylation of Unc-51, like autophagy activating kinase 1; *p-AMPK*, phosphorylation of AMP-activated protein kinase; *NF-κB*, nuclear factor κappa-light-chain-enhancer of activated B cells.

### 4.1 UDCA derivatives

The novel derivative HS-1030 derived from UDCA impeded hepatocellular carcinoma and breast cancer cell growth by inducing apoptosis (Park et al., 1997). Similarly, HS-1183, HS-1199 and HS-1200 generated from UDCA, inhibited proliferation of acute myeloid leukemia by inducing apoptotic cell death by downregulation of caspase-3/8 (Choi et al., 2001). Accordingly, these three derivatives inhibited human prostate carcinoma proliferation due to apoptosis induction via arresting cell cycle progression (Choi et al., 2003). In human cervical carcinoma cells, HS-1183, HS-1199, and HS-1200 suppressed cell growth and induced apoptosis by activating the JNK and NF-κB signaling pathways (Im et al., 2005). Moreover, all of HS-1030, HS-1183, HS-1199, and HS-1200 displayed the ability to inhibit colon cancer cell growth by arresting cell cycle progression at the G1 phase (Park et al., 2004). Finally, HS-1183, HS-1199 and HS-1200 derivatives not only inhibited breast carcinoma cell proliferation in a dose-dependent method, but also induced apoptotic nuclear changes and sub-G1 population and DNA fragmentation through ap53-independent pathway (Im et al., 2001).

In recent studies, CDCA and UDCA were conjugated with the anticancer drug paclitaxel (PTX) via a high-yield condensation reaction. The resulting product chenodeoxycholic-PTX hybrid (CDC-PTX) displayed comparable cytotoxicity and cytoselectivity to PTX. This activity was distinct from the ursodeoxycholic-PTX hybrid (UDC-PTX) that displayed limited anticancer effects on only colon cancer cells (Melloni et al., 2022).

### 4.2 CDCA derivatives

CDCA derivatives HS-1199 and HS-1200 induced caspase-dependent apoptosis in gastric cancer cell lines. This activity was also found dependent on elevated orphan receptor Nur77 (TR3) (Jeong et al., 2003). HS-1200 demonstrated an anticancer effect on human hepatoma cells as it reduced expression levels of cyclin A/D1 and Cdk2 and upregulated p21 WAF1/CIP1 and p27 KIP1 in a p53-dependent manner. HS-1200 also decreased cyclooxygenase (COX)-2 levels and induced early expression of Egr-1 (Park S. E. et al., 2008). In line with these findings, HS-1200 showed potential to induce apoptosis of hepatocellular carcinoma (HCC) (Liu et al., 2008). HS-1200 sensitized human breast carcinoma cells to radiation-induced apoptosis by increasing Bax expression and translocation into the mitochondria and thus increasing cytochrome c release (Yee et al., 2007). Both HS-1199 and HS-1200 exerted an anticancer effect on malignant GB cells through various apoptotic manifestations, including caspase-3 activation, DNA fragmentation factor (DFF) degradation, poly (ADP-ribose) polymerase cleavage, nuclear condensation, and proteasome activity inhibition (Yee et al., 2005). These two derivatives could induce apoptosis in GC cells through a caspase- and mitochondria-dependent manner (Moon et al., 2004). Treatment of thyroid carcinoma cells with HS-1200 increased cell death accompanied by procaspase-3/7 degradation, ADP-ribose polymerase degradation, histone hyperacetylation and peripheral chromatin condensation (Kim et al., 2009). Compound IIIb inhibited multiple myeloma cell proliferation in a way associated with Mcl-1 and PARP-1 cleavage, NF-κB signaling inhibition and/or DNA fragmentation (El Kihel et al., 2008).

### 4.3 DCA derivatives

DCA-chalcone amides were synthesized and tested for their antitumor effects on human lung and cervical cancer cells. The studies demonstrated that specific synthesized DCA-chalcone conjugates exhibited promising outcomes to inhibit cancer cells as potential anticancer agents (Patel et al., 2022). Recently, a series of new DCA derivatives were synthesized by incorporating aliphatic diamine and amino alcohol or morpholine moieties at the C3 position through 3, 26-epoxide ring-opening reactions. The mechanistic studies demonstrated that compound 9 induced cell death in colon cancer cells by activating apoptosis and autophagy. Vitamin D receptor was the primary target of this compound (Markov et al., 2019).

Considerable efforts were added to investigate the anticancer effects of amino-substituted α-cyanostilbene derivatives and CA and DCA amides on the human osteosarcoma (HOS) cancer cells. These studies revealed that all CA α-cyanostilbene amides exhibited anticancer effects on HOS cells with an effective range from 2 to 13 μM through induction of apoptotic cascade (Agarwal et al., 2018).

A pH-responsive micellar hydrogel system was developed using DCA-micelle (DCA-Mic) and carboxymethyl chitosan hydrogel (CMC Hyd) to improve the effectiveness of 5-FU against skin cancer and minimize side effects. This system facilitated the delivery of 5-FU into the skin and exhibited enhanced anticancer activity against melanoma cell growth compared to 5-FU alone. The 5-FU@Mic-Hyd platform showed a promising delivery system with improved efficacy for managing skin cancer in the absence of notable systemic toxicity (Pourmanouchehri et al., 2022).

A conjugate of heparin with DCA exhibited cytostatic and antiangiogenic properties, enhanced the anticancer effects of Doxorubicin (DOX) on squamous cell carcinoma and melanoma cells. Furthermore, the combination treatment using these two drugs resulted in improving therapeutic efficacy while minimizing cytotoxic effects (Park K. et al., 2008).

### 4.4 LCA derivatives

LCA and its derivatives ent-LCA induced apoptosis through CD95 activation, leading to increased ROS generation and subsequent cleavage of procaspase-8 (Katona et al., 2009). A group of BA derivatives, including CA, CDCA, UDCA, and LCA against colon cancer were designed and synthesized. All the compounds exhibited an anti-proliferative signature in various human malignant tumors. Four specific compounds from 4–7 significantly inhibited colon cancer colony formation, migration, and invasion. In addition to their antitumor effects, these compounds induced apoptosis by cell cycle arresting, resulting in a blockage of the mitotic process. Furthermore, they decreased the potential of the mitochondrial membrane but increased intracellular levels of ROS. These compounds downregulated the expression of Bcl-2 and p-STAT3, contributing to their apoptotic and anti-proliferative effects. Interestingly, these compounds also exhibited anti-inflammatory activity by inhibiting the production of nitric oxide (NO) and downregulating the expression of TNF-alpha, both of which are associated with inflammation in colon cancer (Wang et al., 2022).

Using LCA as a basis, ten cationic amphiphiles with variations in their head cationic charged groups were synthesized, and the anticancer effects of these amphiphiles were determined in colon cancer. LCA-based amphiphile containing piperidine head group (LCA-PIP) was approximately 10 times more cytotoxic than its precursor. The enhanced activity of LCA-PIP was attributed to a high level of cellular apoptosis. LCA-PIP induced sub-G0 arrest and caspase cleavage, promoting programmed cell death (Singh et al., 2015).

A heparin-lithocholic conjugate (HL) was created by covalently bonding lithocholate to heparin, and subsequent conjugation with folate to synthesize folate-HL conjugate (FHL). Although HL and FHL showed low anticoagulant activity, they sustained antiangiogenic properties. HL and FHL demonstrated similar antiangiogenic activity and inhibition of proliferation, while FHL exhibited stronger apoptotic effects than HL. These findings highlighted the potential of FHL as an effective anticancer agent with antiangiogenic and apoptotic properties (Yu et al., 2007).

LCA acetate induced leukemia cell differentiation. Combined treatment with LCA acetate and cotylenin A displayed more effectiveness in inducing monocytic differentiation than LCA acetate or cotylenin A alone. LCA acetate activated MAPK signaling that mediates cell differentiation. The synergistic effects of LCA acetate and cotylenin A on cell differentiation were partially ascribed to the MAPK activation induced by both agents (Horie et al., 2008).

### 4.5 CA derivatives

LLC-202, a prodrug for liver cancer, was developed by conjugating oxaliplatin with CA. The conjugation was achieved using 3-NH (2) (−) cyclobutane-1,1-dicarboxylate as a linker between the oxaliplatin analog and the CA moiety. The CA component was firmly bonded to the linker via an amide bond. Compared to oxaliplatin alone, LLC-202 exhibited enhanced absorption by human liver cancer cells while showing less affinity for normal liver cells. LLC-202 possessed higher anticancer activity and efficacy than oxaliplatin through the induction of apoptosis. These findings highlighted the promising potential of LLC-202 as a liver cancer-specific prodrug (Jiang et al., 2023).

A series of BAs (CA and DCA) aryl/heteroaryl amides linked with alpha-amino acid were synthesized and evaluated for the anticancer properties. More specifically, CA derivatives 6a, 6c, and 6m bearing phenyl, benzothiazole, and 4-methyl phenyl groups showed inhibitory activity against breast cancer cells compared with cisplatin and doxorubicin. Meanwhile, 6e, 6i, and 6m exhibited robust activity against the GB cancer cells relative to cisplatin and doxorubicin (Agarwal et al., 2016).

### 4.6 Other bile derivatives

Different BA derivatives were synthesized with modified side chains and the steroid skeleton, in which the former included reaction with 2-amino-2-methylpropanol and 4,4-dimethyl oxazoline group, and cyclization of amides. The latter involved addition of steroid skeleton oxo groups in positions 7 (2, 2a, 2b) and 7,12 (3, 3a, 3b). By Wittig reaction, the ethylidene groups were introduced regio- and stereo-selectively on C-7 and without stereoselectivity on C-3. Compounds containing both C-7 ethylidene and C-12 carbonyl groups (6, 6a, and 6b) showed significant anticancer activity. Altering the carboxylic group to the amide or oxazoline group enhanced cytotoxicity (Bjedov et al., 2017).

A series of new seco-A ring BA diamides were synthesized and evaluated for their anti-proliferative activities. These compounds enhanced G1 arrest and increased anti-migration activity, demonstrating improved anti-proliferative activities relative to the parent bile acid. A compound 27 conjugated with piperazine showed promising results with strong cytotoxicity in cancer cells (Mao et al., 2016). Moreover, all tested compounds exhibited lower cytotoxic activity on noncancerous cells.

Fifteen new piperazinyl bile carboxamides derived from various BAs, including CA, UDCA, CDCA, DCA, and LCA, were synthesized and evaluated for their pro-apoptotic potency in colon cancer cells. Most of the synthetic bile carboxamide derivatives were found to significantly decrease cell viability, in which compound 9c and 9d exhibited the most significant dose-response effect and solubility on colon cancer cells. The presence of a benzyl group in the structure of the derivatives was associated with enhanced anti-proliferative activity. Furthermore, introducing an α-hydroxyl group at the 7-position of the steroid skeleton was particularly beneficial (Brossard et al., 2014).

Two BA tamoxifen conjugates were synthesized using LCA, DCA, and CA, whereby1, 2, or 3 tamoxifen molecules were attached to the hydroxyl groups of BAs with free acid and amine functionalities in their tail regions. In these conjugates, the cholic acid-tamoxifen conjugate with a free amine headgroup (CA-Tam3-Am) demonstrated the strongest potency as an anticancer agent to induce apoptosis, cell cycle arrest, and high ROS generation. These findings highlighted that BAs could be utilized as a new framework to achieve high effective drug potency. The antitumor properties of these conjugates were significantly influenced by the charge and hydrophobicity of the lipid-drug conjugate (Sreekanth et al., 2013).

Four cationic bile acid-based facial amphiphiles were synthesized and evaluated for their cytotoxic activities against colon cancer cells. The critical factors examined were charge, hydration, and hydrophobicity. Among the synthesized amphiphiles, the singly charged amphiphile based on lithocholic acid (LCA-TMA1) exhibited the highest cytotoxicity. In contrast, the triply charged cationic amphiphile based on cholic acid (CA-TMA3) showed negligible cytotoxicity. These cytotoxic effects were observed at late apoptosis. The LCA-TMA1 amphiphile demonstrated high hydrophobicity combined with a burdensome charge, leading to efficient dehydration and significant membrane perturbations. These characteristics facilitated its translocation and resulted in high cytotoxicity. On the other hand, the highly hydrated and multiple-charged amphiphile CA-TMA3 showed the least membrane penetration, limiting its translocation and subsequent cytotoxicity. Amphiphiles based on deoxycholic acid (DCA-TMA2) and chenodeoxycholic acid (CDCA-TMA2), featuring two charged head groups, displayed intermediate behavior. In conclusion, the charge, hydration, and hydrophobicity of these cationic BA-based facial amphiphiles determined their interaction with cells and membrane translocation (Singh et al., 2013).

Brossard et al. (2010) utilized nitrogenous heterocycles as a fundamental component in synthesizing conjugate BA derivatives. They successfully synthesized new piperazinyl BA derivatives and examined *in vitro* activity in different human cancer cells. Among the synthesized derivatives, N-[4N-cinnamylpiperazin-1-yl]-3alpha,7beta-dihydroxy-5beta-cholan-24-amide (compound 7b) and N-[4N-cinnamyllpiperazin-1-yl]-3alpha,7alpha-dihydroxy-5beta-cholan-24-amide (compound 7c) demonstrated the most significant pro-apoptotic activity in these human cancer cells. These compounds induced nuclear and DNA fragmentation, indicating that 7b and 7c induce cell death through an apoptotic process. The findings suggest hybrid heterocycle-steroid compounds could serve as a new class of anticancer drugs with improved bioactivity. Additionally, the simple synthesis of these compounds highlighted their potential for future development as anticancer therapeutics (Brossard et al., 2010).

Králová et al. (2008) synthesized and utilized conjugates of porphyrin and BAs as ligands to specifically bind to saccharide cancer markers expressed by tumor cells. They found that these compounds possessed a high selectivity for saccharide cancer markers and cancer cells, indicating significant potential for targeted photodynamic therapy (Králová et al., 2008). LCA acetate inhibited hepatoblastoma, colon cancer and leukemia cell proliferation by binding to VDR (Adachi et al., 2005). Moreover, bile-acid-appended triazolyl aryl ketones (6af and 6cf) inhibited breast cancer cell viability (Agarwal et al., 2021).

## 5 Conclusion

This article comprehensively reviews the anticancer activities observed after treatment with both natural BAs and synthetic BA derivatives. These therapeutic approaches are attributed to the amphiphilic nature of BAs and their ability to activate additional targeted pathways that are not stimulated at physiological low concentrations. Additionally, the interaction between BAs and the gut microbiome, known as the BA/gut microbiome axis, may influence the association between BAs and cancer, facilitating BAs action (Song et al., 2020b).

Synthesized BA derivatives have strong ability to induce cell death in various human cancer cell lines. Consequently, these novel BA derivatives show promising results as potent agents to target different types of cancer cells by inducing apoptosis. These findings suggest that these derivatives are the potential candidates for developing novel alternative anticancer agents. Nonetheless, to better understand these agents, mechanistic insights of their activities remain to be substantially investigated. While there is currently no precise report on the cost-effectiveness of preparing BAs and their derivatives, we believe that through further research, the price of isolation, purification or synthesis expanse of BAs and derivatives can be reduced, potentially making it more affordable for a greater number of cancer patients.
